# Submassive Central Saddle and Extensive Bilateral Pulmonary Embolism Presenting as Syncope Treated with Catheter-directed Therapy

**DOI:** 10.5811/cpcem.2017.12.36410

**Published:** 2018-01-18

**Authors:** Jessica Andrusaitis, Mohammad Helmy, Christopher E. McCoy, Wirachin Hoonpongsimanont, Bharath Chakravarthy, Shahram Lotfipour

**Affiliations:** * University of California, Irvine, School of Medicine, Irvine, California; † University of California, Irvine, Department of Radiological Sciences, Orange, California; ‡ University of California, Irvine, Department of Emergency Medicine, Orange, California

## Abstract

Massive and submassive pulmonary emboli (PE) are rare but potentially life-threatening medical conditions that necessitate immediate recognition and appropriate treatment. We report a 52-year-old man who was found to have a submassive central saddle and extensive bilateral PEs after experiencing a syncopal event and who had evidence of right heart strain and pulmonary hypertension. He was subsequently treated with catheter-assisted thrombectomy and pulmonary artery tissue plasminogen activator administration. This case report presents an outcome in a patient who received an innovative therapy that has not been well established in this subset of patients.

## INTRODUCTION

Pulmonary embolism (PE) remains a common cause of morbidity and mortality with estimates of up to 200,000 deaths annually.^1^ The two severe forms of this disease are massive and submassive PE. A massive PE is defined by the presence of hemodynamic compromise with a systolic blood pressure <90 millimeters of mercury (mmHg) or a drop in systolic BP ≥40 mmHg from baseline for >15 minutes or hypotension requiring vasopressors not explained by other causes.^2^ Submassive PE is defined by evidence of right ventricular dysfunction with hemodynamic stability.^2^ Although massive PEs are rare and comprise only about 2–5% of all PEs,^3^–^5^ they are crucially important because they carry a 52.4% 90-day mortality rate.^3^ Despite being a rather infamously feared entity, the submassive PE lacks consensus on the optimal evidence-based therapy. This disease is an important entity for physicians to promptly identify and appropriately treat because of the high potential for negative outcomes.

## CASE REPORT

A 52-year-old morbidly obese male presented to the Emergency Department (ED) with a chief complaint of syncope. The morning of presentation he reported feeling lightheaded with shortness of breath and blurry vision upon standing. He next remembered waking up on the floor. He reported worsening shortness of breath over the prior three days. Additionally, he reported experiencing a dull, constant, pressure-like sensation over the left side of his chest that had begun the night before. Initially, the patient denied any medical conditions other than morbid obesity; however, several hours into his stay we learned that he had a history of a deep vein thrombosis (DVT) with a PE after a knee arthroscopy two years earlier.

His temperature was 36.5 degrees Celsius, heart rate of 114 beats per minute, blood pressure of 137/91 mmHg, respiratory rate of 18 breaths per minute, and pulse oximetry of 94% on room air. His weight was 181 kilograms (body mass index of 59). The patient was awake, alert, conversing appropriately, and in no apparent distress. He was noted to be tachycardic but was well perfused, with no signs of cyanosis and a capillary refill less than two seconds. His lungs were clear to auscultation bilaterally without an increase in work of breathing. Other than venous stasis changes, his lower extremities appeared normal.

**Image 1:**
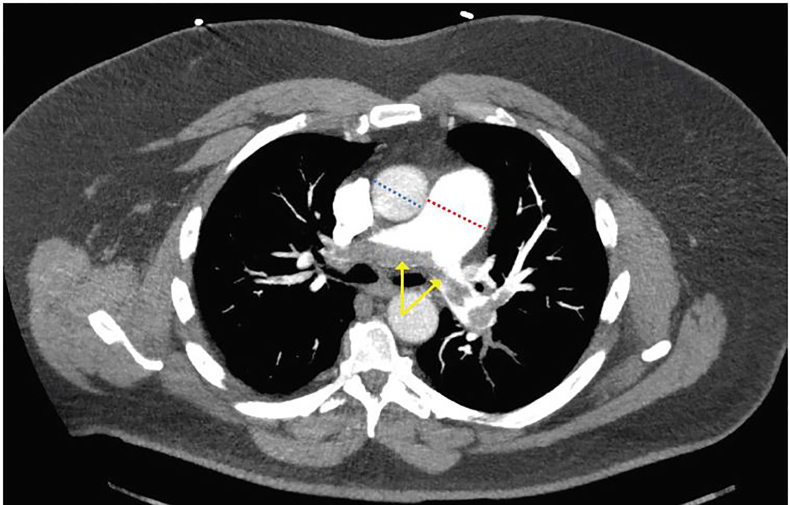
Computed tomography pulmonary angiogram demonstrating a saddle embolism at the main pulmonary artery bifurcation extending into right and left pulmonary arteries (yellow arrows). Also visible is an enlarged main pulmonary artery diameter (red dashed line) relative to the ascending aorta (blue dashed line) indicating right-sided heart strain with pulmonary hypertension.

**Image 2:**
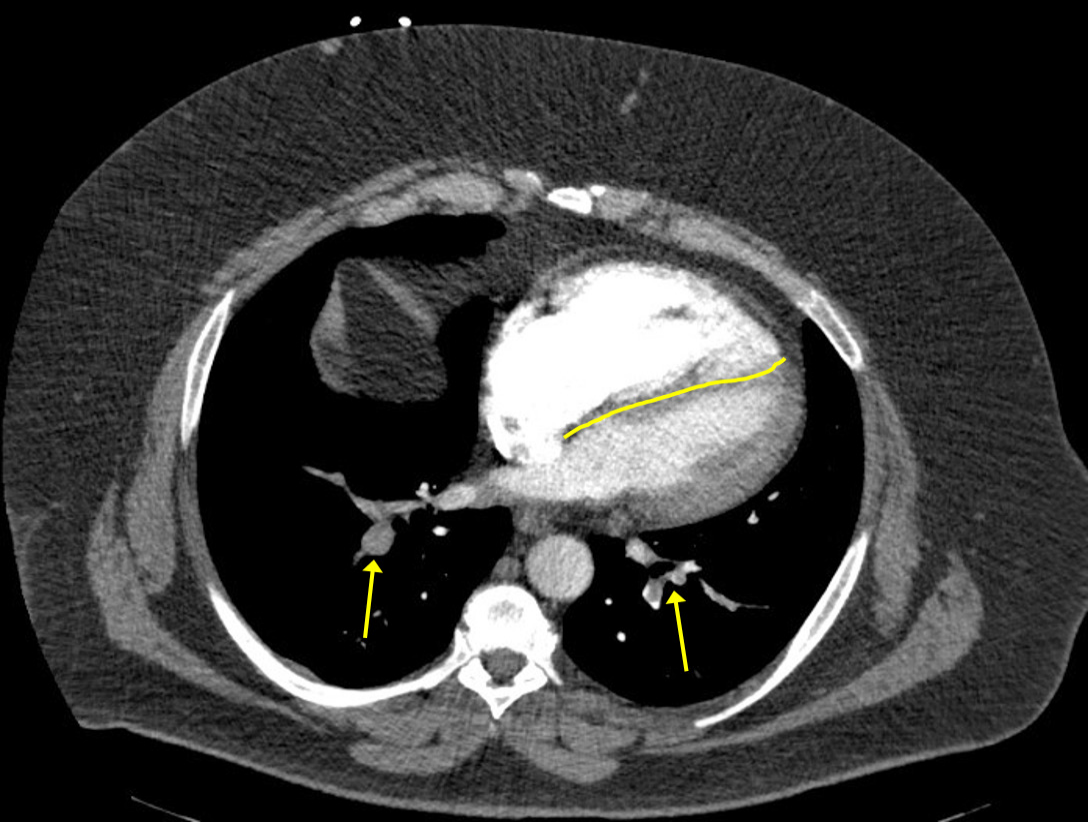
Additional view of the computed tomography pulmonary angiogram demonstrating occlusive and nonocclusive lower lobe pulmonary artery emboli bilaterally (yellow arrows). Straightening and leftward bowing (yellow line) of the interventricular septum indicating right-sided heart strain is also visible.

His electrocardiogram showed sinus tachycardia at 103 beats per minute with a S-wave in lead I, Q-wave in lead III, and small ST-elevations in V1–V3. His initial troponin was above the normal limit at 0.17 ng/mL [0.00–0.03 ng/mL]. The patient’s metabolic panel was notable only for an elevated glucose of 206 mg/dL [85–125mg/dL]. His complete blood count was normal except for an elevated white blood cell count of 12.3 thous/MCL [4–10.5 thous/MCL]. D-dimer was >1000ng/ml [<500 ng/ml]. A computed tomography pulmonary angiogram was ordered to rule out PE (Images 1 and 2). It demonstrated a central saddle embolism and multiple occlusive and nonocclusive lobar, segmental, and subsegmental pulmonary arterial emboli bilaterally. Right heart strain and pulmonary hypertension were evidenced by enlargement of the main pulmonary artery and straightening to leftward bowing of the interventricular septum, indicating significant clot burden.

Pulmonary critical care and interventional radiology teams were consulted and after evaluating the patient, the decision was made to not start heparin and instead immediately take the patient for thrombectomy. The patient underwent clot aspiration and catheter-directed intra-procedural tissue plasminogen activator (tPA) administration into each pulmonary artery. Post-thrombectomy angiogram demonstrated significant improvement, but showed persistent areas of clots. Following the procedure, the patient became hypotensive and hypoxemic requiring vasopressors and continued intubation.

The following day the patient had bilateral pulmonary artery EKOS^TM^ catheters (EkoSonic® Endovascular System designed for the treatment of PE) placed for continuous tPA administration.

On hospital day three, his pulmonary artery angiogram demonstrated no visible thrombus and the EKOS^TM^ catheters were removed. The patient remained in the intensive care unit (ICU) intubated, sedated, and receiving anticoagulants. On hospital day 4, he had sustained hypoxia and then suffered a cardiac arrest but had return of spontaneous circulation after cardiopulmonary resuscitation. This event was thought to be due to a recurrent PE, and thus an inferior vena cava filter was placed the following day.

On hospital day 14, he was extubated and transferred out of the ICU. The patient returned to his baseline mental status without any breathing difficulties or chest pain. On hospital day 20, he was transferred to a skilled nursing facility for rehabilitation. At time of discharge, he was neurologically intact and required minimal assistance with activities of daily living.

## DISCUSSION

PE is a form of venous thromboembolism that is estimated to affect 600,000 patients per year in the United States.^6^ The well-known predisposing factors include recent surgery or trauma, hormone replacement, oral contraceptives, pregnancy, immobility, chemotherapy, and malignancy.^7^–^8^ This patient’s previous PE occurred after a known predisposing factor of arthroscopic knee surgery and resulting immobility. However, his current episode of PE seemed to have no predisposing factors other than obesity and sedentary lifestyle. About 20% of patients with PEs have no identifiable predisposing factors.^9^ PEs typically present with dyspnea (80%), pleuritic chest pain (52%), substernal chest pain (12%), cough (20%), syncope (19%), tachypnea (70%), and tachycardia (26%).^7^ This patient exhibited dyspnea, substernal chest pain, syncope, and tachycardia.

CPC-EM Capsule

What do we already know about this clinical entity?

The etiology, presentation, and risk factors of pulmonary emboli (PE) are well known. There remains a lack of consensus on the recommended treatment for submassive PE.

What makes this presentation of disease reportable?

The presentation of this disease was unique in that the chief complaint was syncope, and shortness of breath was not a prominent finding.

What is the major learning point?

Catheter-directed therapies are increasingly being used for the treatment of submassive PE.

How might this improve emergency medicine practice?

Early recognition of submassive pulmonary embolism and prompt treatment with selected therapy should lead to better outcomes for individuals with PE.

The sequence of pathophysiological events that occur with a PE can be deadly. Initially, an embolus that arises from elsewhere in the body, typically from a DVT in an extremity, lodges in the pulmonary arterial system. Depending on the size of the emboli and the size and abruptness of the resulting occlusion, symptoms can vary dramatically from totally asymptomatic to instant death. It is estimated that more than 30–50% of the arterial bed must be affected before the patient becomes symptomatic.^7^ Once the embolus occludes the vessel, the pulmonary vascular resistance is abruptly increased and the ability of the right ventricle to match this increase in pressure determines if the patient will survive.^7^ If the right ventricle cannot keep up with the increase in pressure, then right ventricular failure will ensue leading to shock and death.

This case is significant for the decision-making that occurred after the diagnosis was made. As it currently stands, there is a lack of consensus in the literature regarding the optimal treatment for the different types of PEs. And indeed, in the case above, there was a brief time of uncertainty in deciding which treatment method was best for this particular patient. Ultimately, he was successfully treated with an innovative approach that has yet to be well established in patients with submassive PEs.

The treatment options for PEs are systemic thrombolytics, anticoagulation, open thrombectomy, and catheter-assisted techniques, including fragmentation and local thrombolytic delivery. In 2004 the American College of Chest Physicians (ACCP) published their evidence-based guidelines for treating venous thromboembolic diseases.^10^ At that time, the recommendation was that patients with massive PEs should receive systemic thrombolytics, and it was “unsettled” whether patients with submassive PEs should receive thrombolytics or anticoagulants. Mechanical approaches and pulmonary embolectomies were recommended only when 1) thrombolytics were contraindicated or failed, and 2) the patient was in critical status.

In the decade or so since those guidelines were published, catheter-directed therapies (CDT) have made small but important strides towards becoming more accepted. This reluctance to embrace CDT has been attributed to the lack of large-scale high-quality trials, lack of an established protocol, requirement for trained personnel and specialized equipment, and a delay in obtaining Food and Drug Administration approval for these techniques and for an intrapulmonary thrombolytic drug.^11^ Some studies have emerged from interventional radiology and vascular publications documenting the success of CDT as treatment for submassive and massive PEs.^3^,^11^,^12^ These studies have documented about an 86% success rate with a 7% rate of complication,^11^–^12^ compared to the old standard of systemic thrombolytics that has about a 77% success rate but comes with a 20% risk of serious hemorrhage.^11^

In 2016 the ACCP published updated guidelines for the treatment of venous thromboembolism.^13^ These guidelines reiterated the 2004 recommendation that massive PEs should receive systemic thrombolytics, and concluded that submassive PEs should not receive systemic thrombolytics but may warrant aggressive anticoagulation. Lastly, the committee recommended against the use of CDT over systemic thrombolytics and also against catheter fragmentation for massive and submassive PEs, citing low quality of evidence and lack of trials as their rationale for this recommendation. CDT was only recommended in cases in which the patient was hypotensive, thrombolytics could not be given, and appropriate expertise and resources were available.^13^

## CONCLUSION

The value of this case is that it demonstrates how our patient was successfully treated with an innovative approach that has yet to be well established in patients with submassive PEs. The intention of this report was to provide a small data point in an area awaiting higher quality evidence and large trials. While awaiting those results, it is the responsibility of the treating physician to select a therapy option that is best suited to the clinical status of the individual PE patient.
